# Socioeconomic inequalities in lifestyle risk factors across low- and middle-income countries

**DOI:** 10.1186/s12889-021-11014-1

**Published:** 2021-05-20

**Authors:** Charlotte Dieteren, Igna Bonfrer

**Affiliations:** Erasmus University Rotterdam, Erasmus School of Health Policy & Management, P.O. Box 1738, 3000 DR Rotterdam, The Netherlands

**Keywords:** Lifestyle, Tobacco, Overweight, Alcohol, Low- and middle-income countries, Concentration index, Socioeconomic inequality, Multilevel analysis

## Abstract

**Background:**

The heavy and ever rising burden of non-communicable diseases (NCDs) in low- and middle-income countries (LMICs) warrants interventions to reduce their underlying risk factors, which are often linked to lifestyles. To effectively supplement nationwide policies with targeted interventions, it is important to know how these risk factors are distributed across socioeconomic segments of populations in LMICs. This study quantifies the prevalence and socioeconomic inequalities in lifestyle risk factors in LMICs, to identify policy priorities conducive to the Sustainable Development Goal of a one third reduction in deaths from NCDs by 2030.

**Methods:**

Data from 1,278,624 adult respondents to Demographic & Health Surveys across 22 LMICs between 2013 and 2018 are used to estimate crude prevalence rates and socioeconomic inequalities in tobacco use, overweight, harmful alcohol use and the clustering of these three in a household. Inequalities are measured by a concentration index and correlated with the percentage of GDP spent on health. We estimate a multilevel model to examine associations of individual characteristics with the different lifestyle risk factors.

**Results:**

The prevalence of tobacco use among men ranges from 59.6% (Armenia) to 6.6% (Nigeria). The highest level of overweight among women is 83.7% (Egypt) while this is less than 12% in Burundi, Chad and Timor-Leste. 82.5% of women in Burundi report that their partner is “often or sometimes drunk” compared to 1.3% in Gambia. Tobacco use is concentrated among the poor, except for the low share of men smoking in Nigeria. Overweight, however, is concentrated among the better off, especially in Tanzania and Zimbabwe (Erreygers Index (EI) 0.227 and 0.232). Harmful alcohol use is more concentrated among the better off in Nigeria (EI 0.127), while Chad, Rwanda and Togo show an unequal pro-poor distribution (EI respectively − 0.147, − 0.210, − 0.266). Cambodia exhibits the largest socioeconomic inequality in unhealthy household behaviour (EI − 0.253). The multilevel analyses confirm that in LMICs, tobacco and alcohol use are largely concentrated among the poor, while overweight is concentrated among the better-off. The associations between the share of GDP spent on health and the socioeconomical distribution of lifestyle factors are multidirectional.

**Conclusions:**

This study emphasizes the importance of lifestyle risk factors in LMICs and the socioeconomic variation therein. Given the different socioeconomic patterns in lifestyle risk factors - overweight patters in LMICs differ considerably from those in high income countries- tailored interventions towards specific high-risk populations are warranted to supplement nationwide policies.

**Supplementary Information:**

The online version contains supplementary material available at 10.1186/s12889-021-11014-1.

## Background

The heavy and ever rising burden of non-communicable diseases (NCDs) in low- and middle-income countries (LMICs) warrants interventions to reduce their underlying risk factors, which are often linked to lifestyles. To effectively supplement nationwide policies with targeted interventions, it is important to know how these risk factors are distributed across socioeconomic segments of populations in LMICs.

An estimated 73% of total deaths worldwide are attributable to NCDs [1]. In LMICs NCD-related deaths are expected to increase from about 30 million currently to 41.8 million by 2030 [2]. The Sustainable Development Goal agenda [3] aims to reduce pre-mature mortality from NCDs with one-third by 2030. Progress so far has been uneven, both across and within countries [4], and the COVID-19 pandemic is eroding earlier gains [5].

The links between lifestyle risk factors or preventable factors - tobacco use, harmful alcohol use and the combination of unhealthy diet and physical inactivity resulting in overweight - and NCDs are well documented [6–8]. In high income settings, lifestyle risk factors are most prevalent among those with a lower socioeconomic status [9, 10]. Less is known about socioeconomic inequalities in lifestyle risk factors across LMICs [11], which limits opportunities for targeting effective interventions on those exposed to greatest risk which is an approach that is increasingly being implemented in among others healthcare facilities [12, 13]. Such targeted interventions could provide an important supplement to national policies such as taxation of unhealthy foods and tobacco to reduce consumption of these goods. An important, and sometimes underappreciated aspect of these lifestyle risk factors, is the limited choice that individuals might have in adopting and changing these unhealthy lifestyles due to structural inequalities. Overweight for example, is in many cases not simply a result of a choice to consume unhealthy foods, but a result of a food environment with limited food options available [14].

Yaya et al. [15] (2018) studied women across 33 Sub Saharan African countries and found that alcohol consumption and overweight were more prevalent among the better off, while tobacco use was more concentrated in the poor segments of the population. A systematic review by Allen et al. (2017) [16] on the association between socioeconomic status and lifestyle risk factors in LMICs, included data from 75 studies conducted between 1990 and 2015. Only two studies [17, 18] reported data on more than one LMIC. Hosseinpoor et al. (2012) [17] studied 48 LMICs and found that daily smoking as well as low fruit and vegetable consumption were more prevalent among those with a lower socioeconomic status. However, their data are dated and lack information on alcohol consumption and overweight. In addition, unhealthy behaviours are likely to cluster [19], and there is not much known about the prevalence of multiple lifestyle risk factors within a person or household in LMICs. In a comment on Allen et al. (2017) [16], Stringhini & Bovet (2017) [20] emphasize the lack of systematically compared data from LMICs to determine and explain socioeconomic inequality in lifestyle risk factors.

This study aims to fill part of that gap by quantifying the prevalence and socioeconomic inequalities in three lifestyle risk factors - smoking, harmful alcohol use and overweight -in 9 low-income countries (LICs) and 13 middle-income countries (MICs). This will help to identify policy priorities conducive to the Sustainable Development Goal of a one third reduction in deaths from NCDs by 2030. In this study, we use self-reported information on smoking, while overweight is determined by actual measured height and weight. Insights about harmful alcohol use are based on reports from a randomly selected subsample of women about alcohol use of their partner. We also extend the existing literature by studying cumulation of these unhealthy behaviours within households to determine which couples are most likely to jointly exhibit two or more of these three lifestyle risk factors.

We use data from 1,278,624 adults in the Demographics and Health Surveys (DHS) between 2013 and 2018. We investigate whether countries that spend more on health are less likely to exhibit large socioeconomic inequalities in lifestyle risk factors, which would be expected if several well targeted prevention programs are in place. However, it seems more likely that the better off are the first to benefit from early prevention programs, if available. We then estimate a multilevel model to associate individual characteristics with tobacco use, overweight, harmful alcohol use and the cumulation of these lifestyle risk factors within a household.

## Methods

### Data

We exploit the Standard Demographic and Health Surveys (DHS) containing data from nationally representative, randomly selected samples of women in reproductive age (15–49) and smaller samples of randomly selected men (age 15–59) in LMICs [21]. The DHS is comparable across countries and contains data on demographics and health behaviour. The DHS is publicly available for research purposes and the corresponding research protocols are reviewed and approved by an Institutional Review Board. Informed consent is obtained from each respondent before the start of the interview [22].

We include all Demographic and Health Surveys from 2013 onwards containing data on at least one of three unhealthy lifestyles: tobacco use, overweight and harmful alcohol use. Unfortunately, the data do not allow us to disentangle overweight into aspects related to unhealthy diet versus physical inactivity, but we are referring to the combination of these two when studying “overweight”. Surveys collected before 2013 are excluded to ensure policy relevance. In case more than one DHS was fielded in the same country since 2013, we include the most recent one. This results in data from 9 LICs and 13 MICs covering a total of 1,029,182 women, as shown in Supplementary Table S1. Sample sizes range from 6116 (Armenia) to 699,686 (India) women. Most countries were African (*n* = 16), a few Asian (*n* = 5) and one Eastern European. Interviews with men were in most countries performed in every third household [18] resulting in a total sample of 249,442 men from 19 countries, with country-level sample sizes ranging from 2755 (Armenia) to 112,122 (India) men as shown in Supplementary Table S1. No data were collected among men in Chad, Egypt and Tajikistan. To estimate cumulative presence of lifestyle risk factors within a household, we use data from the subset of couples where both partners were interviewed in respectively the female and male survey. Sample sizes for each of the four outcome measures are shown in Table S1. Overweight (*n* = 882,820), based on height and weight measured during the interview is missing for 14% of women. Pregnant women were excluded from data collection on height and weight since BMI is not perceived an accurate measure of body composition during pregnancy [23]. Other women did not consent to have their measures taken. Harmful alcohol use is based on women’s reports about alcohol consumption of their partner and was only collected among a randomly selected subsample of women via the “domestic violence” module of the DHS [18], resulting in a considerably smaller sample (*n* = 184,381). Tobacco use among men was available for virtually the entire sample (0.01% missing) and information from both partners was available for 125,393 couples.

Tobacco use was extremely low among women (ranging from only 0.1% in Tanzania to 5.2% in Rwanda) so we only included tobacco use among men. No data on husband or partner alcohol use was available for Albania, as shown in Table S1. In Egypt and Gambia less than 2 % of women indicated that their husband or partner consumed alcohol. Consequently, Albania, Egypt and Gambia are excluded in the estimation of socioeconomic inequality in husband/partner’s harmful alcohol consumption.

In addition to these micro level data, we use the most recent macro level information (2017) on country health expenditure as percentage of GDP [24], to proxy nationwide investments in health.

### Variables of interest

The three lifestyle risk factors are operationalized as follows. *Tobacco use* is based on self-reported information among men about current smoking, i.e. daily, sometimes or never. Data on local smoking products were also collected but were excluded due to heterogeneity and low response rates. For ease of interpretation, tobacco use is dichotomized with 1 representing “daily” or “sometimes” and 0 otherwise. Secondly, the Body Mass Index (BMI) based on weight and height measured during the interview by trained surveyors, is used to define the dichotomous variable *overweight* with a BMI larger than 25.0 identified as overweight [25] and 0 otherwise. Height and weight measurements were only obtained among women, not among men. Thirdly, a randomly selected subset of women was asked whether their husband or partner consumed alcohol (*alcohol use)* and if yes, whether their husband or partner was never, sometimes or often drunk. Responses indicating that the partner was sometimes or often drunk were considered *harmful alcohol use* [1] and 0 otherwise. In addition to these three lifestyle risk factors at individual level, we also study the cumulation of these factors at household level. The cumulative presence of the three lifestyle risk factors was estimated within a household, using the couples dataset. Household behavior was dichotomized into 1 *unhealthy* with two or the maximum of three lifestyle risk factors present in the couple, and 0 otherwise (e.g. non or one lifestyle risk factor).

To proxy socioeconomic status, we use the wealth quintiles provided in the DHS. These are derived from a wealth index ranking households based on a principal component analysis on a set of variables about household dwelling characteristics and asset ownership [26] including materials used for construction of the house, types of water access, sanitation facilities and ownership of televisions and bicycles [27]. This composite measure is considered an acceptable measure of wealth in LMICs. The lowest wealth quintile represents the 20% poorest part of the population while the highest quintile reflects the 20% best off. Filmer & Pritchett (2001) [26] have shown that that this method provides plausible and defensible weights for an asset index to serve as a proxy for wealth, even though income or expenditure data are not taken into account. To acknowledge the complexity and multidimensionality of socioeconomic status we also include education and occupation in our regression analysis.

Macrolevel country health expenditure is defined as the share of GDP spent on health i.e. *health expenditure as % of GDP* in 2020, the most recent data available [24]. We use health expenditure for all countries in our study sample for the same year, as opposed to the different data collection years, to allow for easier comparison. Ideally, we would have obtained information on spending earmarked for prevention and/or NCDs, but these are not available, to our knowledge.

Our multilevel models to determine individual characteristics of those using tobacco, being overweight, using harmful levels of alcohol and the cumulation of these in couples include rural/urban household, age categories, literacy, education, occupation, wealth index, household size and number of living children.

### Analytical approach

#### Prevalence of lifestyle risk factors

The prevalence of the lifestyle risk factors for each country is calculated as a crude prevalence rate: dividing the total number of respondents with the lifestyle risk factor by the total number of respondents in the relevant study sample. For household unhealthy behaviour the presence of two or three lifestyle risk factors in couples over the total number of couples was used as the prevalence.

#### Socioeconomic inequalities

We measure socioeconomic inequalities in lifestyle risk factors, i.e. variation in these factors across the wealth quintiles, with a concentration index suggested by Erreygers (2009) [28] for a dichotomous variable. This is simply the scaled covariance between the lifestyle risk factor (*y*_*i*_) of individual *i* and their (fractional) rank (*R*_*i*_) in the distribution of the wealth index:

1 $$ EI(y)=8\ \mathit{\operatorname{cov}}\left({y}_i,{R}_i\right) $$

This index takes values between − 1 and 1. Positive values indicate a disproportionate concentration of the lifestyle risk factor among the better off, while negative values signal a disproportionate concentration among the poor. The STATA package “conindex” [29] is used for this analysis.

The micro level analyses to estimate prevalence and socioeconomic inequalities in lifestyle risk factors (*tobacco use, overweight, harmful alcohol use and unhealthy household behaviour*) are performed separately for each country.

#### Macro level association between inequalities and health expenditures

The within country estimations of inequalities are followed by a cross-country macro level analysis determining whether countries with smaller inequalities also tend to be those with a higher percentage of GDP spent on health. We calculate the average percentage of GDP spent on health across the countries in our sample as a reference value. Any country’s spending on health above the reference value is considered “high” and otherwise “low”. We map each country, differentiating between low- and middle- income countries, on a four-quadrant model of socioeconomic inequality (vertical axis) and health expenditure (horizontal axis) to identify countries with a “double disadvantage” i.e. both a skewed distribution of lifestyle risk factors towards the poor and low spending on health nationwide. This allows for identification of countries that could be prioritized by policy makers worldwide.

#### Multilevel analysis

We then estimate a multilevel probit model based on country fixed effects for each of the four (cumulative) lifestyle risk factors using the pooled set of data from all countries included in the study. Eq. 2 describes the multilevel model.

2 $$ {y}_{ic}={\beta}^{\prime }{x}_i+{\gamma}_c+{\varepsilon}_{ic} $$

where *x*_*i*_ is the set of individual characteristics, *γ*_*c*_ the country fixed effects and *ε*_*ic*_ the error term. This allows us to estimate the relation between individual characteristics and lifestyle risk factors while allowing for variation in these across countries.

All analyses were performed using software package STATA 15.

## Results

### Prevalence of lifestyle risk factors

Table S1 in the Supplementary files shows for each country the response rate on the lifestyle risk factors. The prevalence of the lifestyle risk factors by country (Table 1) indicate that tobacco use is most prevalent in Armenia and Timor-Leste: respectively 59.6 and 54.4% of men reported to smoke daily or sometimes. The highest level of overweight among women is found in Egypt (83.7%), followed by South Africa (61.6%) and Albania (58.5%). There is a wide range in the prevalence of women that reported that their partner is “often or sometimes drunk”, with only 1.3% in Gambia and 82.5% in Burundi. In total, the prevalence was over 50% in three countries (Burundi, Cambodia and Rwanda). Only four countries had a prevalence below the 20% (Ethiopia, Gambia, Sierra Leone and Tajikistan), all low-income countries. The prevalence of cumulative unhealthy behaviour within households (at least two lifestyle risk factors in a couple) is also diverse. Armenia and South Africa have the highest prevalence of unhealthy household behaviour with 50.6 and 40.4%.

**Table 1:** Prevalence of lifestyle risk factors

Country [code]	Income level	Tobacco use men		Overweight women		Harmful alcohol women’s spouse		Unhealthy household behaviour	
		**N (%)**		**N (%)**		**N (%)**		**N (%)**	
**Albania** [ALB]	MIC	2077	(33.8)	8617	(58.5)	*na*		*na*	
**Armenia** [ARM]	MIC	1641	(59.6)	2609	(45.5)	1358	(38.4)	715	(50.6)
**Burundi** [BDI]	LIC	1003	(12.2)	713	(9.0)	4081	(55.4)	280	(14.3)
**Cambodia** [KHM]	MIC	1652	(31.8)	2007	(18.6)	2885	(82.5)	871	(35.8)
**Chad** [TCD]	LIC	*na*		1066	(11.0)	825	*(21.7)*	*na*	
**Congo** [CO]	MIC	1837	(21.2)	1149	(14.1)	2291	(40.4)	106	(6.4)
**Egypt** [EGT]	MIC	*na*		16,197	(83.7)	*na*		*na*	
**Ethiopia** [ETH]	LIC	1189	(9.7)	1579	(11.5)	873	(18.5)	140	(4.5)
**Gambia** [GMB]	LIC	787	(20.6)	915	(21.9)	47	(1.3)	70	(8.0)
**India** [IND]	MIC	17,412	(15.5)	120,983	(18.5)	19,581	(29.7)	6892	(13.5)
**Kenya** [KEN]	MIC	2175	(17.0)	3959	(29.4)	1518	(33.6)	660	(19.1)
**Malawi** [MWI]	LIC	941	(12.6)	1621	(21.9)	1619	(30.0)	492	(15.2)
**Nigeria** [NGA]	MIC	1144	(6.6)	8380	(24.7)	3523	(15.9)	503	(8.1)
**Rwanda** [RWA]	LIC	627	(10.1)	1361	(21.9)	980	(51.5)	153	(16.9)
**Sierra Leone** [SLE]	LIC	2002	(27.6)	1372	(18.8)	660	(15.4)	225	(12.2)
**South Africa** [ZAF]	MIC	1381	(38.2)	2008	(61.6)	1716	(43.0)	237	(40.4)
**Tajikistan** [TJK]	LIC	*na*		3658	(36.9)	927	*(17.5)*	*na*	
**Tanzania** [TZA]	MIC	448	(12.8)	3378	(28.1)	2176	(28.6)	207	(16.3)
**Timor-Leste** [TLS]	MIC	2512	(54.4)	1094	(9.3)	1243	(33.7)	163	(11.9)
**Togo** [TGO]	LIC	466	(10.4)	1194	(27.2)	1525	(28.4)	232	(12.9)
**Zambia** [ZMB]	MIC	3014	(20.4)	3210	(21.6)	4270	(45.4)	1598	(25.9)
**Zimbabwe** [ZWE]	MIC	1553	(18.5)	3307	(36.5)	2230	(38.5)	882	(28.8)

### Inequalities in the distribution of lifestyle risk factors

Table 2 shows the socioeconomic inequalities, based on the Erreygers Index (EI), for each of the lifestyle risk factors. Tobacco use is in all countries concentrated among the poor, except for Nigeria where we found a pro-rich distribution. Overweight, on the other hand, is concentrated in all countries towards those with a higher socioeconomic status. Armenia is the only country without significant inequality in overweight across socioeconomic groups. Socioeconomic inequalities in harmful alcohol use is more diverse. Four countries (Kenya, Sierra Leone, South Africa and Zimbabwe) show no wealth related inequalities in alcohol consumption. Nigeria is the only country were harmful alcohol use is most concentrated among the better off (EI 0.127), while Chad, India, Rwanda and Togo show the strongest unequal distribution towards the poor (EI respectively − 0.147, − 0.139, − 0.210, − 0.266). Accordingly, the Erreygers Indices for unhealthy household behaviour show a diverse picture. Unhealthy behaviour within a household is most concentrated among the poor (EI − 0.253) in Cambodia. Only in Ethiopia, India, Nigeria and Timor-Leste significant positive Erreygers indices are reported, referring to a concentration among the better off (EI respectively 0.033, 0.063, 0.090 and 0.101).

**Table 2:** Erreygers Indices estimates for lifestyle risk factors and national health care expenditures

Country	Income level	Erreygers Index – lifestyle risk factors				Health care expenditure^**1**^	
		Tobacco	Overweight	Harmful alcohol partner/husband	Unhealthy household behaviour	% of GDP	∆ of μ % GDP
**Albania**	MI	−0.011	*0.034*	*na*	*na*	6.7	0.7
**Armenia**	MI	− 0.011	0.009	*− 0.062*	− 0.004	10.4	4.4
**Burundi**	LI	*−0.147*	*0.073*	*−0.191*	*− 0.071*	7.5	1.5
**Cambodia**	MI	*−0.206*	*0.053*	*−0.049*	*− 0.254*	5.9	− 0.1
**Chad**	LI	*Na*	*0.061*	*− 0.141*	*Na*	4.5	−1.5
**Congo**	MI	*−0.145*	*0.074*	*−0.088*	0.017	2.9	−3.1
**Egypt**	MI	*Na*	*0.069*	*na*	*Na*	5.3	−0.7
**Ethiopia**	LI	*−0.045*	*0.150*	*0.082*	*0.033*	3.5	−2.5
**Gambia**	LI	*−0.028*	*0.072*	*Na*	0.029	3.3	−2.7
**India**	MI	*0.049*	*0.210*	*−0.139*	*0.063*	3.5	−2.5
**Kenya**	MI	*−0.093*	*0.127*	0.005	0.010	4.8	−1.2
**Malawi**	LI	*−0.012*	*0.060*	*−0.045*	− 0.021	9.7	3.7
**Nigeria**	MI	*0.072*	*0.169*	*0.127*	*0.090*	3.8	−2.2
**Rwanda**	LI	*−0.042*	*0.094*	*−0.210*	*− 0.071*	6.6	0.6
**Sierra Leona**	LI	*−0.230*	*0.058*	0.014	0.025	13.4	7.4
**South Africa**	MI	*−0.053*	*0.157*	0.000	−0.038	8.1	2.1
**Tajikistan**	LI	*Na*	*0.071*	*−0.024*	*Na*	7.2	1.2
**Tanzania**	MI	*−0.053*	*0.227*	*−0.113*	−0.016	3.7	−2.3
**Timor-Leste**	MI	*−0.115*	*0.083*	*−0.112*	*0.101*	3.9	−2.1
**Togo**	LI	*−0.091*	*0.144*	*−0.266*	*−0.040*	6.2	0.2
**Zambia**	MI	*−0.188*	*0.179*	*−0.089*	*−0.096*	4.5	−1.5
**Zimbabwe**	MI	*−0.097*	*0.232*	−0.011	*−0.062*	6.6	0.6
***Average***						6.0	

^1^ Rounded based on two decimals with ∆ of μ % indicating how a country’s GDP spend on health differs (Δ) from the average over all countries (μ) in percentage points

*Italian: significant differences between wealth quintiles p < 0.05*

The last column of Table 2 shows how the country average share of GDP spend on health differs (Δ) from the total average share (μ) in percentage points, indicating whether a country spends more or less than the average (6%) across these 22 LMICs. Sierra Leone spends the highest share of GDP on health (13.4%), followed by Armenia (10.4%) and Malawi (9.7%). On the other end of the spectrum, Congo, Gambia and India spend the lowest share of GDP on health, respectively 2.9, 3.3 and 3.5%.

### Socioeconomic inequalities and percentage of GDP spent on health

**Fig. 1:**
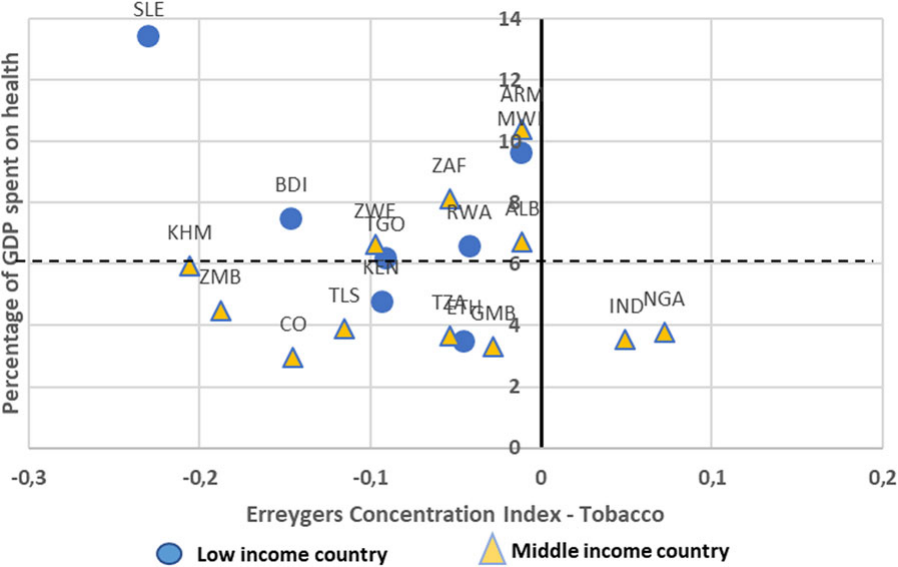
Tobacco use EI indices and percentage of GDP spent on health (with reference line for average % of GDP spent)

**Fig. 2:**
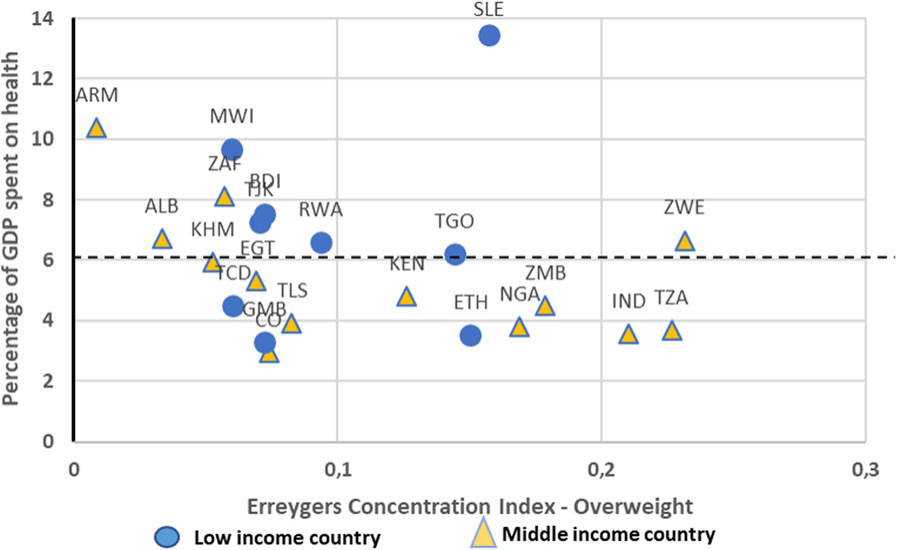
Overweight EI indices and percentage of GDP spent on health (with reference line for average % of GDP spent)

**Fig. 3:**
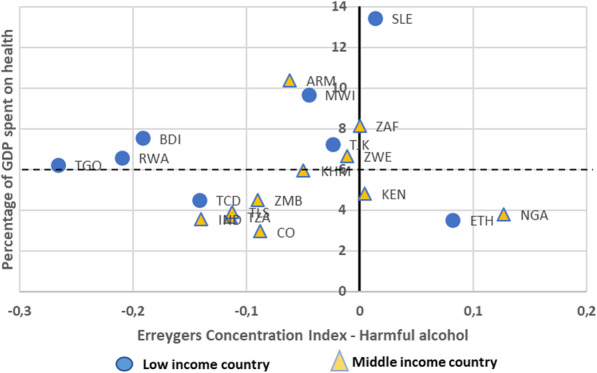
Harmful alcohol use EI indices and percentage of GDP spent on health (with reference line for average % of GDP spent)

**Fig. 4:**
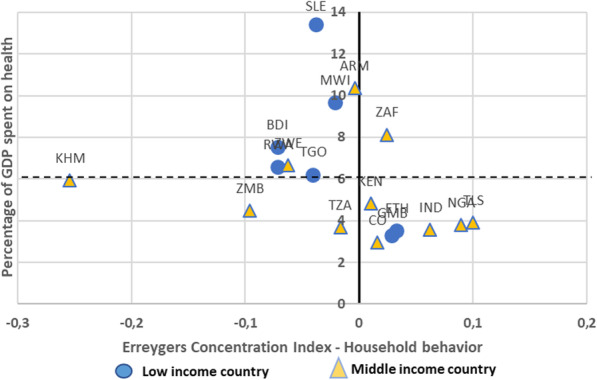
Unhealthy household behavior EI indices and percentage of GDP spent on health (with reference line for average % of GDP spent)

Figures 1 to 4 map each country on our four-quadrant model with socioeconomic inequality in the lifestyle risk factor of interest on the horizontal axis and average percentage of GDP spent on health on the vertical axis, distinguishing between low- and middle-income countries. The dotted reference line represents the average share of GDP (6.0%) spent on health across the countries in our sample. The top left quadrant contains countries with relatively higher shares of health expenditure, but the corresponding lifestyle risk factor concentrated among the poor where the latter is generally deemed “unfair”. The top right quadrant consists again of countries with relatively higher shares of health expenditure, but the lifestyle risk factor is concentrated among the better-off, which might suggest that these countries do not have the highest priority when it comes to reducing lifestyle risk factors among the poor, even though there is still considerable room for improvement. The bottom right quadrant shows low shares spend on health and concentration of the lifestyle risk factor among the better off. Finally, the bottom left corner contains the countries with a “double disadvantaged” population with the share spend on health relatively low and lifestyle risk factors concentrated among the poor. Countries in this bottom left quadrant should be prioritized in policies to reduce the morbidity and mortality from NCDs.

Tobacco use is mainly concentrated in the left quadrants (Fig. 1), so among the poor (conventionally labelled as: “pro-poor”). This disadvantage is especially present for middle-income countries, where the pro-poor inequality coincides with relatively low shares of GDP spent on health. While we cannot identify an overall pattern it seems that, with the exemption of Sierra Leone, a higher share of GDP spent on health is observed in countries with less inequality i.e. an concentration index closer to zero. We observe for overweight (Fig. 2), contrary to tobacco use, a pro-rich distribution with countries spending a higher share of their GDP on health having less inequality.

When mapping these countries in terms of harmful alcohol use (Fig. 3), there are countries located in each of the four quadrants indicating that there is no strong association between the share of GDP spent on health and the socioeconomical distribution of harmful alcohol use. However, the countries at a “double disadvantaged” (bottom left quadrant) and thus with the most pressing need for effective interventions to reduce harmful alcohol use are Chad, Congo, India, Tanzania, Timor-Leste, Zambia and Cambodia. Figure 4 shows clustering of lifestyle risk factors in households (couples) and suggests that countries with a higher share of their GDP spent on health tend to have a pro-poor distribution of lifestyle risk factors within a household. Countries with a lower share of their GDP spent on health tend to have a pro-rich distribution of lifestyle risk factors within a household. As this figure shows the cumulative behaviour of a household, this finding should be interpreted carefully as combinations of lifestyle risk factors within a household might differ. When comparing low- versus middle-income countries, we observe that the low-income countries more frequently have a pro-poor distribution, while the middle-income countries dominantly have a pro-rich distribution of these lifestyle risk factors. We refrain from multilevel analysis here, because of the limited sample size (*n* = 22 countries).

### Individual characteristics and lifestyle risk factors

Table 3 contains descriptive statistics of the explanatory variables used in the multilevel models on the women’s, men’s and couples’ samples. The information in Table 3 is based on the men and women in the couples’ dataset.^1^ For all countries combined, we find that a bit less than one third live in an urban area (30.1%) and half of the women indicate not to have an occupation (49.7%) compared to 4.7% among men. The average age in our sample is 37 years with an average household consisting of six members.

**Table 3:** Means of explanatory variables in multilevel probit regression model based on the couples’ dataset^1^

	Female		Male	
	N (%)		N (%)	
Urban household	37,702	(30.1)	37,702	(30.1)
Literate	75,446	(60.2)	96,423	(77.0)
Secondary/higher educated	51,985	(41.4)	73,423	(58.6)
No occupation	61,727	(49.7)	5838	(4.7)
White-collar	4867	(3.9)	12,333	(9.9)
Blue-collar	22,308	(18.0)	48,524	(39.1)
Agriculture	35,342	(28.5)	57,325	(46.2)
	**Mean (std. dev)**		**Mean (std. dev)**	
Age	37.2	(8.1)	37.3	(8.6)
Household size	5.8	(2.9)	5.8	(2.9)
Number of living children	2.8	(1.9)	3.1	(2.5)
N	125,393		125,393	

The results from the multilevel probit regression models (Table 4) confirm that tobacco and harmful alcohol use are concentrated among the poorer men, while overweight is concentrated among the better off women. When combining unhealthy behaviors among couples, as shown in the last column, we find that the probability of such clustering significantly increases with 0.07 among couples with women above 35 years old, compared to similar couples with a woman below 20 years old.

**Table 4:** Multilevel probit regression models, average marginal effects, [95% CI]

	Tobacco^**1**^	Overweight^**2**^	Harmful alcohol use^**3**^	Unhealthy household behavior^**4**^
*Rural household*	Ref	Ref	Ref	Ref
Urban household	*0.02 [.02–.03]*	*0.02 [.02–.03]*	*0.03 [.02–.03]*	*0.02 [.02–.03]*
*Age below 20 yrs (F)*		Ref	Ref	Ref
20–35 yrs. (F)		*0.17 [.16–.17]*	*0.09 [.08–.11]*	0.06 *[.04–.08]*
above 35 yrs. (F)		*0.25 [.25–.26]*	*0.11 [.10–.13]*	*0.07 [.05–.09]*
*Age below 20 yrs (M)*	Ref			Ref
20–35 yrs. (M)	*0.16 [.15–.16]*			0.03 [−.03–.09]
above 35 yrs. (M)	*0.17 [.16–.17]*			0.06 [−.00–.12]
*Illiterate (F)*		Ref	Ref	Ref
Literate (F)		*0.03 [.02–.03]*	*0.02 [.02–.03]*	0.02 [.01–.03]
*Illiterate (M)*	Ref			Ref
Literate (M)	0.00 [−.00–.01]			0.01 [−.00–.01]
*Education: Less then primary (F)*		Ref	Ref	Ref
Secondary or higher (F)		*−0.02 [−.02--.01]*	*− 0.01 [−.02--.00]*	0.01
*Education: Less then primary (M)*	Ref			Ref
Secondary or higher (M)	*−0.02 [−.03–.01]*			*− 0.01 [−.02 - -.00]*
*Occupation: none (F)*		Ref	Ref	Ref
white-collar (F)		*0.03 [.02–.03]*	*0.04 [.03–.05]*	− 0.01 [−.02–.00]
blue-collar (F)		*0.01 [.01–.02]*	*0.09 [.09–.10]*	*0.02 [.01–.02]*
agriculture (F)		*−0.03 [−.04--.03]*	*0.11 [.10–.11]*	0.02 [.01–.02]
*Occupation: none (M)*	Ref			Ref
white-collar (M)	*0.02 [.01–.02]*			*− 0.02 [−.04 - -.01]*
blue-collar (M)	*0.06 [.05–.06]*			−0.00 [−.01–.01]
agriculture (M)	*0.04 [.03–.04]*			*− 0.02 [−.03 - -.00]*
Wealth index: very poor	Ref	Ref	Ref	Ref
poor	−0.00 [−.01–.00]	*0.03 [.02–.03]*	*− 0.03 [−.04--.06]*	0.00 [−.00–.01]
middle	*− 0.00 [−.0–.00]*	*0.06 [.06–.07]*	*− 0.05 [−.06- -.05]*	*0.02 [.01–.02]*
rich	*− 0.02 [.03–.02]*	*0.11 [.11–.12]*	*− 0.07 [−.08 - -.07]*	*0.02 [.01–.03]*
very rich	*− 0.05 [−.06--.05]*	*0.17 [.17–.18]*	*− 0.11 [−.12- -.10]*	0.00 [−.01–.01]
Household size	*− 0.00 [−.00--.00]*	*− 0.00 [−.00--.00]*	*− 0.01 [−.01 - -.01]*	*− 0.01 [.01 - -.01]*
Number of living children (F)		*0.00 [.00–.00]*	*0.01 [.01–.01]*	*0.01 [.00–.01]*
Number of living children (M)	*0.00[.00–.00]*			0.00 [−.00–.00]
*Armenia*	Ref	Ref	Ref	Ref
Albania	*−0.17 [−.18--.15]*	0.01 [−.09–.02]	.	.
Burundi	*− 0.32 [−.33--.30]*	*0.14 [.13–.16]*	*0.10 [.08–.12]*	− 0.24 [−.26 - -.21]
Cambodia	*−0.17 [−.18--.15]*	*0.02 [.01–.04]*	*0.40 [.38–.42]*	−0.08 [−.10 - -.06]
Chad	*.*	*0.07 [.05–.08]*	*− 0.16 [−.18 -. -.14]*	.
Congo	*−0.25 [−.26--.23]*	*0.20 [.18–.21]*	*−0.02 [.04 - -.00]*	− 0.34 [−.37 - -.31]
Egypt	*.*	*0.42 [.41–.44]*	.	.
Ethiopia	*−0.38 [−.39--.36]*	*− 0.28 [−.29–.26]*	*−0.21 [−.23 - -.19]*	*−0.37 [−.39 - -.35]*
Gambia	*−0.23 [−.24--.21]*	*0.28 [−.27–.30]*	Sample too small	*−0.28 [−.31 - -.25]*
India	*−0.30 [−.31--.28]*	*−0.24 [−.26--.23]*	*−0.07 [−.08 - -.05]*	*−0.24 [−.26 - -.23]*
Kenya	*−0.29 [−.31--.28]*	*−0.12 [−.13--.10]*	*−0.08 [−.10 - -.06]*	*−0.21 [−.22 - -.19]*
Malawi	*−0.33 [−.34--.31]*	*0.34 [.32–.35]*	*−0.11 [−.13 - -.09]*	*−0.22 [−.25 - -.20]*
Nigeria	*−0.42 [−.43--.40]*	*−0.13 [−.14--.11]*	*−0.26 [−.28 - -.25]*	*−0.31 [−.33 - -.29]*
Rwanda	*−0.37 [.39--.35]*	*0.19 [.18–.21]*	*0.05 [.03–.08]*	*−0.22 [.25 - -.20]*
Sierra Leone	*−0.19 [−.20–.18]*	*0.22 [.21–.24]*	*−0.28 [−.31 - -.26]*	*−0.24 [−.26 - -.22]*
South Africa	*−0.12 [−.13--.10]*	*0.46 [.44–.48]*	*0.04 [.02–.06]*	*−0.07 [−.10 - -.04]*
Tanzania	*−0.32 [−.34--.31]*	*−0.12 [−.13--.10]*	*−0.14 [−.16 - -.12]*	*−0.22 [−.25 - -.19]*
Tajikistan	.	*−0.06[−.08--.05]*	*− 0.19 [−.21 - -.17]*	.
Timor-Leste	−0.00 [−.02–.01]	*−0.38[−.39--.36]*	*−0.04 [.06 - -.02]*	*− 0.26 [−.28 - -.23]*
Togo	*− 0.36 [−.38--.35]*	*0.22 [.20–.23]*	*−0.14 [−.16 - -.11]*	*−0.25 [−.27 - -.22]*
Zambia	*−0.25 [−.27--.24]*	*−0.18 [−.20--.17]*	*0.04 [.02–.06]*	*−0.14 [−.16 - -.12]*
Zimbabwe	*−0.26[−.27--.24]*	*−0.07[−.08--.05]*	*−0.00 [−.02–.01]*	*−0.13 [−.15 - -.11]*
N	245,835	443,528	172,450	90,578

^1^: Men’s dataset; ^2^: Women’s dataset; ^3^: Women’s dataset (reporting on partner’s alcohol use); ^4^: Couples’ dataset

*Italian: significant at p < 0.05*

F: reported in women’s dataset

M: reported in men’s dataset

## Discussion

This study exploits data from the Demographic and Health Survey (DHS) collected across 22 low- and middle-income countries (LMICs) between 2013 and 2018 to analyse prevalence and socioeconomic inequality in lifestyle risk factors i.e. tobacco use, overweight and harmful alcohol use. We show that both the prevalence and the degree of socioeconomic inequality differ considerably across lifestyle risk factors and across countries. Tobacco and harmful alcohol use are largely concentrated among the poor, while overweight is heavily concentrated among the better-off in LMICs. This is contrary to findings from high income countries where all lifestyle risk factors are most prevalent among those with a lower socioeconomic status [9, 10].

The largest socioeconomic inequalities across the four lifestyle risk factors were found for overweight. In developed countries low socioeconomic status is consistently associated with higher prevalence of unhealthy BMI, while our finding shows the opposite direction, in line with previous research on LMICs [17]. The largest pro-rich socioeconomic inequalities in overweight were observed in India, Tanzania and Zimbabwe. Some evidence suggests that obesity is a symbol of high social status in developing countries [30]. This contrast in socioeconomic inequality between high- versus low- and middle-income countries emphasizes the need to develop context specific policy interventions to tackle lifestyle risk factors. For most LMICs in our study, we find that harmful alcohol use is mostly a problem among the poor.

When mapping the share of GDP spend on health in each of these countries, as well as the socioeconomic inequality in lifestyle risk factors, we identified those countries with both a low share spend on health and an inequality lifestyle risk factors distributed towards the poor as “double disadvantaged”. Combining these different lifestyle risk factors, especially Zambia, Tanzania and Cambodia are at a double disadvantage and should be prioritized when implementing global policies to reduce unhealthy lifestyles among the poor.

### Limitations

This study is based on cross-sectional data solely allowing us to examine associations. Our findings cannot be interpreted as causal. Furthermore, our findings are derived from large datasets and inferences about the nature of individuals cannot be deduced from inferences about the larger groups to which these individuals belong i.e. ecological fallacy. The response rates for the DHS are generally high, ranging from 97 to 99.9%. However, missing observations on lifestyle risk factors (Supplementary file S1) because respondents were unwilling to answer or to have their height and weight measured are a limitation to our study. This would be especially problematic when respondents from a specific socioeconomic group are less likely to provide this information, biasing our inequality estimates. However, we do not have reason to believe that this is the case. The sample of women reporting on their partner’s harmful alcohol use does not suffer from this potential bias, because the sample size is lower as a result of only a randomly selected subset of women being asked to answer this question. A second limitation arises from the use of BMI as a combined proxy for diet and physical activity which were not observed in the DHS. Although BMI is an objective and reliable measure it is less informative on diet composition; we cannot identify whether the poor consume less fruit and vegetables. Measuring physical activity, both work and leisure related, would be informative to policy makers aiming to reduce overweight but the DHS does not collect this information. Furthermore, because of data limitations we could not provide information on overweight in men. A third limitation arises from the reports on alcohol use in men, which are provided by the spouse and not by the consumer himself. This is likely to bias our estimates, but it is unclear whether this leads to an over- or underestimation. When alcohol consumption is collected from the consumer himself, it is possible that people who drink excessively provide an under estimation because of a perceived social stigma. However, the spouse might also under report for the same reason and her recall bias might be larger. She could also overestimate her partner’s alcohol use because she does not have complete information on his alcohol consumption, especially not when this is outside of the house. Finally, socioeconomic inequality in lifestyle risk factors could be influenced by taxation policies of which the effects could differ per country and per socioeconomic group. Further research is needed to estimate the effect of such taxation schemes on the socioeconomic distribution of lifestyle risk factors. Notwithstanding these limitations, this study provides policy relevant insights into lifestyle risk factors in LMICs based on data from over a million of adults living in more than twenty LMICs.

## Conclusions

This study emphasizes the importance of lifestyle risk factors in LMICs and the socioeconomic variation therein. While tobacco and alcohol use are most prevalent among males with a low socioeconomic status, it is mainly the better off females that are overweight. We identified Zambia, Tanzania and Cambodia as the countries at a “double disadvantage”, implying that priority should be given to these populations when implementing policies towards the SDG target of reducing NCDs by one third. Given the different socioeconomic distribution of lifestyle risk factors, especially overweight, the targeting of interventions to reduce the burden from these lifestyles in LMICs should not be copied from high income countries but be tailored towards the high-risk populations in these countries. Below we suggest three policy implications.

### Policy implications

Consistent with findings from Yaya et al. (2018) [15] among women across 33 Sub Saharan African countries, we find that tobacco use in men is most prevalent among the poor in LMICs. For HICs, increasing the price of cigarettes has been shown to be one of the most effective strategies to reduce smoking prevalence, in particular among people with a lower socioeconomic status [31]. The World Health Organization’s Framework Convention on Tobacco Control (WHO FCTC) covers more than 90% of the world’s population and provides its 180 collaborators to enact comprehensive, effective tobacco control measures. The findings of this study emphasize that in particular Timor-Leste, Armenia and South Africa which have the highest prevalence in tobacco use can benefit from alignment with the WHO FCTC. Second, to our knowledge, no interventions have been proven to be widely effective in sustainably reducing overweight in high income countries. So even when interventions developed in HICs, such as low caloric diets or physical activity programs, would be tailored to target the better off women in LMICs it is unlikely that these are effective. Furthermore, the food environment can limit the opportunity for the poor to switch to healthy, often more expensive, foods. As a result, overweight is not simply driven by the choice to consume unhealthy foods but can be a reflection of structural inequalities. Further research is therefore necessary to identify effective interventions to reduce overweight in LMICs. Finally, while lifestyle risk factors are generally deemed to be modifiable, support to improve lifestyles is necessary to make sustained changes. Access to this support, if even available, is likely to be smaller for those with a lower socioeconomic status. Targeting this segment of the population, for example through vouchers for support programmes or cash transfers conditional on improving behaviour are therefore likely to be most effective in reducing socioeconomic inequalities in lifestyle risk factors. Further research is necessary to determine the effectiveness of these policy suggestions, especially through a longitudinal approach to identify modifications in unhealthy behaviours over time.

## Supplementary Information

**Additional file 1.**

## Data Availability

The data used in this study are freely available from the DHS website after registration (https://dhsprogram.com/data/available-datasets.cfm).

## Abbreviations

LMIC: Low- and Middle-Income Countries
EI: Erreygers Index
NCD: Non-communicable diseases

## Footnotes

1 Descriptive statistics based on the women and men datasets are available from the authors upon request.
