# Supplementary material for: Socioeconomic inequalities in lifestyle risk factors across low- and middle-income countries
Source: BMC Public Health. 2021 May 20;21:951. doi: 10.1186/s12889-021-11014-1 (PMC8134821; doi:10.1186/s12889-021-11014-1)
Supplement: Supplementary file 1 — Additional file 1. [file 12889_2021_11014_MOESM1_ESM.docx]

# Supplementary files

Table S1. Survey year and sample size for each lifestyle risk factor by country

|  | | | **Men’s data** | | **Women’s data** | | | **Couples’ data** | |
| --- | --- | --- | --- | --- | --- | --- | --- | --- | --- |
| **Country** | **Year survey conducted** | | **Total** | **Tobacco** | **Total** | **Overweight** | **Harmful alcohol use** | **Total** | **Unhealthy household behavior** |
| Albania | | 2017-18 | 6,142 | 6,142 | 15,000 | 14,442 | *na* | *na* | *na* |
| Armenia | | 2015-16 | 2,755 | 2,755 | 6,116 | 5,730 | 3,538 | 1,490 | 1,413 |
| Burundi | | 2016-17 | 7,552 | 7,552 | 17,269 | 7,908 | 7,366 | 3,599 | 1,961 |
| Cambodia | | 2014 | 5,190 | 5,190 | 17,578 | 10,818 | 3,497 | 3,060 | 2,435 |
| Chad | | 2014-15 | *na* | *na* | 17,719 | 9,730 | 3,803 | *na* | *na* |
| Congo DR | | 2013-14 | 8,656 | 8,656 | 18,827 | 8,159 | 5,686 | 4,486 | 1,648 |
| Egypt | | 2014 | *na* | *na* | 21,762 | 19,345 | 6,667 | *na* | *na* |
| Ethiopia | | 2016 | 12,688 | 12,688 | 15,683 | 13,781 | 4,720 | 6,141 | 3,124 |
| Gambia | | 2013 | 3,821 | 3,819 | 10,233 | 4,176 | 3,534 | 1,388 | 878 |
| India | | 2015-16 | 112,122 | 112,122 | 699,686 | 655,156 | 66,013 | 63,696 | 51,060 |
| Kenya | | 2014 | 12,819 | 12,815 | 14,741 | 13,455 | 4,514 | 5,265 | 3,458 |
| Malawi | | 2015-16 | 7,478 | 7,478 | 24,562 | 7,407 | 5,406 | 3,806 | 3,234 |
| Nigeria | | 2013 | 17,359 | 17,344 | 38,948 | 33,894 | 22,247 | 8,658 | 6,212 |
| Rwanda | | 2014-15 | 6,217 | 6,217 | 13,497 | 6,217 | 1,907 | 2,904 | 906 |
| Sierra Leone | | 2013 | 7,262 | 7,258 | 16,658 | 3,261 | 3,993 | 3,725 | 1,848 |
| South Africa | | 2016 | 3,618 | 3,618 | 8,514 | 7,305 | 4,303 | 663 | 587 |
| Tajikistan | | 2017 | *na* | *na* | 10,718 | 9,918 | 5,313 | *na* | *na* |
| Tanzania | | 2015-16 | 3,514 | 3,514 | 13,266 | 12,027 | 7,597 | 1,564 | 1,269 |
| Timor-Leste | | 2016 | 4,622 | 4,622 | 12,607 | 11,805 | 3,694 | 1,981 | 1,369 |
| Togo | | 2013-14 | 4,476 | 4,474 | 9,480 | 4,395 | 5,373 | 2,270 | 1,796 |
| Zambia | | 2013-14 | 14,750 | 14,772 | 16,381 | 14,833 | 9,410 | 7,198 | 6,183 |
| Zimbabwe | | 2015 | 8,401 | 8,396 | 9,937 | 9,058 | 5,800 | 3,499 | 3,064 |
| **Total** | |  | 249,442 | 249,432 | 1,029,182 | 882,820 | 184,381 | 125,393 | 92,445 |
